# Blood and Cancer: Cancer Stem Cells as Origin of Hematopoietic Cells in Solid Tumor Microenvironments

**DOI:** 10.3390/cells9051293

**Published:** 2020-05-22

**Authors:** Ghmkin Hassan, Masaharu Seno

**Affiliations:** 1 Graduate School of Interdisciplinary Science and Engineering in Health Systems, Okayama University, Okayama 700-8530, Japan; pthz2c4o@s.okayama-u.ac.jp; 2 Department of Microbiology and Biochemistry, Faculty of Pharmacy, Damascus University, Damascus 10769, Syria

**Keywords:** tumor microenvironments, hematopoietic cells, cancer stem cells

## Abstract

The concepts of hematopoiesis and the generation of blood and immune cells from hematopoietic stem cells are some steady concepts in the field of hematology. However, the knowledge of hematopoietic cells arising from solid tumor cancer stem cells is novel. In the solid tumor microenvironment, hematopoietic cells play pivotal roles in tumor growth and progression. Recent studies have reported that solid tumor cancer cells or cancer stem cells could differentiate into hematopoietic cells. Here, we discuss efforts and research that focused on the presence of hematopoietic cells in tumor microenvironments. We also discuss hematopoiesis from solid tumor cancer stem cells and clarify the notion of differentiation of solid tumor cancer stem cells into non-cancer hematopoietic stem cells.

## 1. Introduction

**Figure 1:**
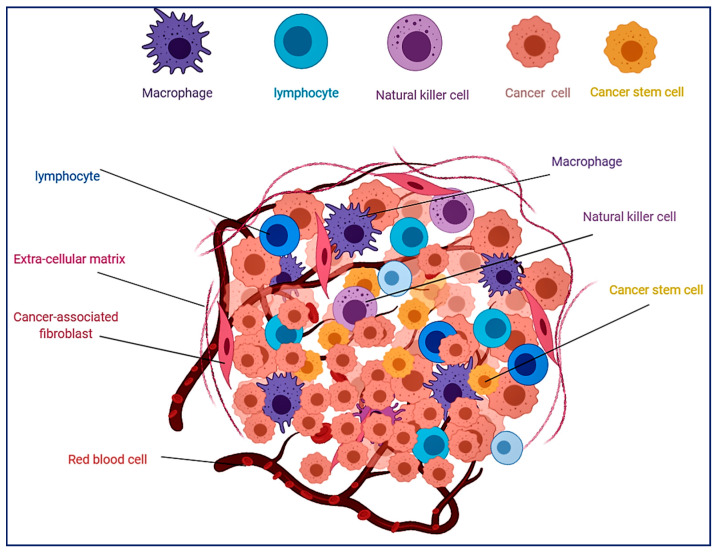
Schematic illustration of tumor microenvironment showing different cell phenotypes including different hematopoietic cells.

The origins of blood and immune cells differ depending on the stage of development. Hematopoietic stem cells (HSCs) residing in the bone marrow or fetal liver have self-renewal ability and differentiation capacity to form all the blood cell lineages [1]. Different hematopoietic cells play different roles in the tumor microenvironment (TME) [2,3,4,5]. These cells either suppress or support tumor growth [6,7,8]. After the tumor occurs, a network of blood vessels surround and try to penetrate the tumor mass through angiogenesis in an attempt to provide nutrients to cancer cells [9,10,11]. The tumor mass has a complex structure and is composed of different types of non-transformed cells, cancer cells, and extracellular matrix components, collectively known as the TME [12,13]. The TME provides unique features for the tumor such as chemotherapy resistance, hypoxia environment, cancer invasion, and metastasis (Figure 1). In addition to growth factors and interleukins, the TME provides other signals that stimulate or induce tumor cells [14,15]. The changes in the TME can alter the signals and interactions between the TME components and, as a consequence, the characteristics of tumors; growth, metastasis, and treatment response may change and affect patient survival [16,17,18]. Tumor hypoxia occurs when oxygen and nutrition become limiting factors in tumor areas due to cell proliferation by blocking the blood supply to the tumor mass [19,20,21]. Under hypoxia conditions, the tumor cells unleash response programs to restore oxygen levels via multiple mechanisms such as angiogenesis induction, metabolic reprogramming, and shifting of antitumor macrophage to tumor-associated macrophages (TAMs) [22,23,24]. Tumor-initiating cells, also known as cancer stem cells (CSCs), are a subpopulation of tumor cells residing in tumor bulk and are capable of self-renewal and differentiation, which provide the ability to rebuild tumor mass and metastasis to other sites [25]. CSCs can respond to tumor microenvironment changes and compounds secreted or produced by non-transformed cells, which could change the CSCs fate and cause differentiation just like other types of stem cells; however, the understanding of this CSC differentiation ability is still unclear [26,27]. CSCs can produce different cell phenotypes such as fibroblasts and endothelial cells, which support growth and recurrence of the tumor through the production and secretion of growth factors and extracellular matrix components in addition to triggering angiogenesis process [28,29,30]. Blood and immune cells exist in the TME of solid tumors and play vital roles in tumorigenesis. Recent studies showed that these cells are not imperatively derived from circulating blood cells or bone marrow hematopoietic stem cells but could have an embryonic origin. Macrophages, lymphocytes, and myeloid-derived suppressor cells (MDSCs) are abundant in most types of cancers [7,31,32]. The existence of these cells can have either positive or negative effects on tumorigenesis and may be associated with a good or poor prognosis depending on their type [6,8]. Accordingly, the available information is changing regarding the fate and origin of cells residing in the TME.

In this review, we summarize different types of hematopoietic cells in the TME of solid cancer. We discuss the recent efforts examining CSCs as one of the possible origins of hematopoietic cells.

## 2. Cancer Stem Cells

Cancer stem cell theory suggests the existence of a cell subpopulation within tumor bulk that has the ability to repopulate and initiate tumors. This self-renewal ability provides a basic and discriminate characteristic that gives CSCs tumorigenicity ability and the capacity to produce heterogeneous cell phenotypes [25]. CSCs can form new tumors when a small number are injected into immunocompromised animal models. When in vitro, they form spheres in low adherent culture conditions; non-CSCs fail to form tumors or spheres under the same conditions. CSCs express stemness markers just like stem cells, including Nanog, Oct3/4, and Sox2, in addition to other surface markers that are considered specific markers for CSCs, such as CD133, CD44, CD24, and EpCAM [33,34]. CSCs have been detected and isolated from many solid cancers such as breast, liver, pancreas, prostate, ovarian, and lung. CSCs are linked to aggressive tumor phenotype, metastasis capacity, and resistance to drugs [25]. In the TME, the microenvironment that surrounds the CSCs creates a CSC niche, which plays a vital role in maintaining CSC stemness. CSC niche cells produce many compounds affecting CSC fate and could stimulate cancer cell invasion and metastasis. CSCs can also initiate metastasis or may differentiate into other cancer cell phenotypes that have metastasis ability [26,28,35].

Normal stem cells that reside in different types of tissue in a dormant state respond to injuries and inflammatory signals, turning stem cells onto the active state [35]. This activation leads to proliferation and cell divisions that could be either symmetric or asymmetric. Asymmetric divisions produce transition progenitors that respond to microenvironment signals and unleash differentiation pathways to repair and regenerate injured tissue parts. Thus, stem cells have the ability to differentiate into various cell phenotypes. This ability differs from one stem cell type to another [36]. Consistent with the same notion, CSCs have the same differentiation potential as stem cells. The boundaries of this ability are still under investigation. Recent studies have confirmed some of the differentiation patterns of CSCs. These differentiated cells were found to play essential roles in tumor progression. Cancer-associated fibroblasts and endothelial cells are two examples of these cells driven from CSCs. In the TME, endothelial cells driven from CSCs trigger the angiogenesis process in an attempt to vascularize the tumor mass, whereas cancer-associated fibroblasts support CSCs by secretion chemokines and growth factors [29,37]. These cells can also communicate with other cells in the TME. These communications could stimulate or alter many signaling pathways in CSCs. The activation of specific signaling pathways could be responsible for directing tumor growth and their response to treatment [26,35].

Purification of CSCs from patient samples, in almost all cases, requires antibodies against specific surfaces markers or using specific culture conditions to promote the enrichment of CSC populations. These methods, therefore, require identification of CSC-specific markers that are not available or adequate in many types of cancers. Thus, CSC purification is still considered a challenging and demanding procedure [38]. CSC enrichment from cell lines does not always represent their counterparts in the primary tumors due to alterations and modifications like secondary genomic changes caused by prolonged culture [39]. Therefore, we established novel CSC models in our laboratory by converting induced pluripotent stem cells (iPSCs) into CSCs in the presence of conditioned media (CM) from cancer cell lines. The CM contain many cytokines, chemokines, and growth factors that direct this conversion without genetic manipulation of iPSCs. We succeeded in establishing different models using CM from lung, breast, pancreas, and liver cancer cell lines. These CSC models were found to be tumorigenic, express stemness and cancer stem cell markers, have differentiation potential, and induce metastasis [25,29,40,41]. iPSC-derived CSC models provide unique tools for studying tumorigenesis mechanisms, pathways, and differentiation capacity according to CSC theory under various conditions that cancer cell lines might fail to cover. Tracking CSC fate in vitro and in vivo could be easier using these models than using other types of cancer cells.

## 3. Hematopoietic Cells in the Microenvironment of Solid Tumors

**Figure 2:**
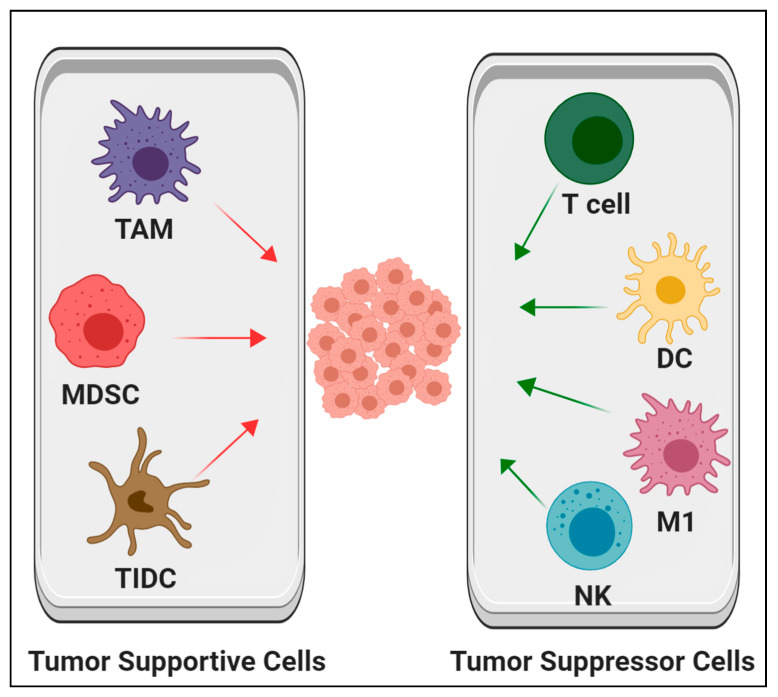
Schematic illustration of some types of hematopoietic cells in the tumor microenvironment that either support or suppress tumor growth. TAM, tumor-associated macrophages; MDSC, myeloid-derived suppressor cells; TIDC, tumor-infiltrating dendritic cell; T cells, T lymphocyte; DC, dendritic cell; M1, M1 macrophage; NK, natural killer cell.

Different types of immune cells exist in the TME and have a critical impact on tumor progression. In normal tissue, immune cells provide defense responses, whereas in the TME, the functions of these cells principally depend on cancer stage, interaction with other cells, and their subtypes [2,4,8,42]. Chronic immune responses can create a chronic inflammatory environment, which is widely thought to be one of the stimulants of carcinogenesis. Nonetheless, many immune suppresser cells have been detected in the TME [43]. Consequently, hematopoietic cells in the TME could either eradicate or support cancer cells (Figure 2). The activation status of many types of immune cells can change in the TME and turn into tumor supporters. The following are the predominant types of hematopoietic cells in the TME.

### 3.1. Tumor-Associated Macrophage

Macrophages in the TME affect tumor growth, survival, the immune response, and angiogenesis. The macrophage phenotype can change depending on the microenvironment. In the TME, macrophages respond to different types of signals and stimulants produced by a wide range of cells and, as a result, switch their phenotype to TAMs [44,45,46]. DeNardo et al. showed that T-cells and tumor cells produce IL-4, which acts as an activator of TAMs. TAMs activated by IL-4 can promote malignancy and metastasis [47]. The increase in cathepsin activity in TAMs is also linked to cancer invasion and tumor angiogenesis during the development of pancreatic and mammary cancer [48]. Instead of triggering an immune response against tumor cells, TAMs suppress the immune response and support the growth and metastasis of the tumor. TAMs effects are basically due to anti-inflammatory cytokines such as IL-10, transforming growth factor-beta (TGF-β), and other secreted factors like basic fibroblast growth factor (bFGF), vascular endothelial growth factor (VEGF), and prostaglandin E2 [49]. Therefore, TAMs enhance tumor metastasis, invasion, and angiogenesis. TAMs also have prognostic value as they are associated with poor survival rates [50,51,52]. The interaction between TAMs and other types of the cells in the TME is mediated by secreted factors by cancer cells and fibroblasts like M-CSF, CCL2, and CCL5. This interaction may result in the recruitment of other cells like myeloid progenitor cells to the TME. The accumulation of TAMs in the TME is linked to chemotherapy resistance in liver, breast, lung, ovarian, and prostate cancers [53].

### 3.2. Tumor-Infiltrating Lymphocytes

In addition to macrophages, lymphocytes are detected in the TME and are recognized as one of the solid tumor TME components. Immune filtrating lymphocytes, also known as tumor-infiltrating lymphocytes (TILs), have been studied recently to understand their functions and roles in human tumor immunity [54,55,56]. These cells affect the immune response toward the tumor and therefore could play roles in tumor aggressiveness, metastasis, and treatment response potential [57,58,59].

Even though almost all studies attempted to link the presence of TILs with survival rate and response to different types of cancer treatments, the origin of these cells was supposed to be bone marrow [60]. Although the presence of TILs was thought to have a positive effect on survival and prognosis, several studies could not find this connection. As such, for macrophages, different types of lymphocytes were proposed to be linked to different functions. Regulatory T-cells showed to support tumor growth, whereas other T-cells showed the potential for reducing metastasis and recurrence of tumors. CD4-positive T-helper cells could either support or quash the progression of solid tumors [50,61,62]. Notably, CD3+, CD4, and CD8+ TILs were shown to secret pro-inflammatory cytokines and suppress tumor growth, whereas cancer cells were found to produce CCL18, which increases migration of regulatory T cells to tumor sites and promotes tumor progression. Therefore, the interaction between tumor cells and different leukocyte phenotypes could result in either suppressing or promoting tumor progression [63,64].

### 3.3. Tumor Dendritic Cells and Myeloid-Derived Suppressor Cells

Dendritic cells (DCs), antigen-presenting cells existing in all tissues, affect cancer progression and have been detected in the TME. In general, DCs interact with T-cells and enhance immune responses against cancers [65]. However, cancer cells and the TME can alter DCs function and result in the recruitment of other types of DCs with immune-suppressive characteristics. In the TME, DCs could convert to an immune-suppressive state [66]. Different subsets of DCs have different roles in cancer progression. CD11c+ DCs were shown to promote tumor growth and hasten tumor expansion in the early stage of ovarian cancer development [42]. Tumor-infiltrating dendritic cells (TIDCs), with low expression of co-stimulatory molecules and reduced cross-presenting capacities, improve tumor cell survival through suppression of cytotoxic T-cells [67,68,69].

Finally, MDSCs are considered one of the important types of hematopoietic cells in the TME. MDSCs arise from myeloid lineage and have immunosuppressive ability, and therefore increase tumor growth and making the tumor resistant to immunotherapy [70,71]. Cancer-cells-derived factors, including cytokines, chemokines, growth factors, and metabolites, lead to the accumulation of MDSCs in the TME [72]. Since MDSCs are heterogeneous cells, different markers have been applied to identify their subtypes. The monocytes subtypes of MDSCs (MO-MDSCs) showed severe immunosuppressive effects in the TME, whereas another subtype, called the polymorphonuclear/granulocytic (PMN-MDSCs), demonstrated less immunosuppressive effects than MO-MDSCs [73]. However, not all subtypes of MDSCs are known and their functions have not been fully elucidated.

In the TME, the roles of immune cells in human cancers and their relationship with CSCs are still not completely understood. This field needs more work to address the many questions that could lead to the development of new treatment strategies for solid cancers and the enhancement of immunotherapy effects. The origins of those cells is one of these questions.

## 4. Hematopoiesis from Solid Cancer Stem Cells

Historically, the origin of blood has attracted scientific attention. In the last century, intensive work was conducted in the field of hematology and, as a result, some mysteries about hematopoiesis and different hematopoietic cell functions have been resolved. The identification of hemogenic endothelium as a precursor of hematopoietic cells was reported at the end of the 1800s by some researchers who reported the existence of hematopoietic lineage cells in the endothelium of the embryo’s aorta [74,75]. After this observation, hematology research revealed that the location of hematopoiesis changes throughout life. Hematopoiesis begins with the yolk sac in the early stages of embryo development, then in the liver and spleen, and finally in the bone marrow during adult life. Residing in the bone marrow, HSCs supply the body with different types of hematopoietic cells by differentiation to many stages of progenitor cells initially followed by terminal differentiation. Many details and conceptions regarding signal requirements and regulation mechanisms for hematopoietic stem differentiation have been corrected [76,77]. Simultaneously, new theories were established regarding hematopoiesis and the interaction between different types of cells in the TME as new research tools and techniques were developed. Likewise, some reports suggested that somatic cells such as fibroblasts or endothelial cells can be reprogrammed to hematopoietic-differentiated cells by inducing the expression of specific transcription factors such as PU.1 and cEBPα, which could reprogram cells into macrophage-like cells, whereas LMO2, RUNX1c, SCL, ERG, and GATA2 reprogram them to a hematopoietic fate with erythroid and myeloid potential [78]. The origin of macrophages in the TME is now controversial. Recent studies showed that TAMs have an embryonic origin and they are driven from the yolk sac rather than from circulating monocytes that differentiated from bone marrow HSCs. TAMs of embryonic origin accumulate in the TME along with others of circulation monocyte origin [79,80,81]. The depletion of TAMs of embryonic origin was reported to reduce tumor burden, indicating that these cells play a dominant role in the TME [81]. As mentioned above, CSCs have differentiation potential, which raised the question of the ability of CSCs to differentiate into hematopoietic cells in the TME. If so, what types of hematopoietic cells could arise from CSCs and what are their roles in the TME? Another interesting question is found in the possibility of CSCs to produce hematopoietic progenitor cells with a homing ability to bone marrow.

Recently, more data regarding these questions have become available. Several studies reported that cells from several cancer cell lines can form polyploidy giant cancer cells (PGCCs) with the expression of the stemness marker under hypoxia conditions. These cells can differentiate into erythroid lineage both in vitro and in vivo [82,83,84]. The treatment of breast cancer cell line cells, MCF-7, were reported to generate PGCCs with differentiation capacity into myoepithelial, endothelial, and erythroid cells, in addition to exhibiting cancer stem cell characteristics [85,86]. Thus, these studies demonstrated the ability of cancer cells with stemness characteristics to differentiate into erythroid cells in vitro. However, the functional assays for erythroid cells produced from CSCs are not comprehensive. These data are still preliminary and require further experiments using different types of cancer cells in addition to in vivo studies. Although no profound analysis of the mechanisms or why they occur has been conducted, the phenomena have been noticed and reported. These data form a proof of concept that provides an opportunity for further investigation and discussion.

In the case of prostate cancer, analyzing the transcriptome of disseminated cancer cells obtained from prostate cancer patients with non-metastasis showed the existence of cells derived from prostate cancer in the bone marrow. These cells expressed transcripts such as CD34, CD45, and CD33, which are markers for hematopoietic progenitor or stem cells, in addition to hemoglobin α2, which were detected in more than 90% of analyzed cells [87]. From these data, cancer cells could produce cells with a hematopoietic cell signature and transcriptomes with migration ability to the bone marrow. Consistent with the same notion, immature neutrophils were detected in cancer patient blood circulation. These cells are also thought to arise as a result of the influence of factors secreted from tumor cells [7,88]. Zheng et al. reported that β-globin is selectively deregulated in cancer cells and could contribute to cancer cell survival as a cyto-protective agent, and could even affect their metastasis ability [89]. Hematopoietic cells in the TME can be differentiated by specific gene expression, as shown by Chifman et al. by analyzing microarray expression datasets [90]. Regarding red blood cell hematopoiesis, the ability of cancer cells to generate blood cells positive for fetal hemoglobin (HbF) within solid tumors has been proven [91,92,93]. Fetal hemoglobin supplies the tumor with oxygen and supporting tumor growth. Erythropoiesis targeting was suggested as one of the therapeutic strategies in solid cancer therapy [94]. The HCC1937 cell line cells-derived spheres were reported to express and secrete hemoglobin, suggesting that they act as a protector for cells during oxidative stress [88]. Human glioma PGCCs produced red bodies that confirmed in glioma tissue sections. These bodies were located in and around the cytoplasm of PGCCs and expressed different types of hemoglobin, confirming that they were erythrocytes [95].

Hemogenic endothelial cells, a small population of endothelial cells, are one of the origins of hematopoietic cells [96]. Hematopoietic stem cells can differentiate from the hemogenic endothelium by endothelial to hematopoietic cell transition (EHT). In EHT, hematopoietic stem cells develop by budding from the hemogenic endothelium, where endothelial cells switch to detached and free-moving hematopoietic stem cells. The EHT was thought to be restricted to the early stages of embryo development. However, a recent report has shown that EHT can occur in the bone marrow in the late fetus/young adult [97]. Both blood and endothelial cells have the same origin, mesodermal progenitors, and similar gene expression patterns. Attempts to uncover the origin of hematopoietic cells using lineage tracing methods and mouse models revealed that both endothelial and hematopoietic progenitors have the same origin, hemangioblasts. In the early stages of embryonic development, hematopoietic cells are first producing with limited potency and give maturation signals to secondary hemato-lymphoid territories. This event could also occur in tumor microenvironments where hematopoietic cells could give differentiations to CSCs or maturation signals to hematopoietic progenitor cells driven from CSCs [98]. Transcription factor Runx1 was identified as a specific transcription factor that can distinguish hemogenic endothelium from other endothelial cell types. Runx1 was shown to have an essential function in HSC formation from endothelial cells but its role ends when the Vav gene is expressed [99,100,101,102].

On the other hand, it became clearer that the interaction between hematopoietic cells and vascular endothelium has many mechanisms. In a recent report, Plein et al. showed that erythro-myeloid progenitors could give endothelial cells that contribute to blood vascular formation [103]. Tumor angiogenesis is the formation of new blood vessels within tumor mass in an attempt to supply the tumor with nutrients and oxygen. Tumor angiogenesis involves the emergence of new endothelial cells [104]. In the TME, endothelial cells that form the inner lining of the blood vessels are heterogeneous and affect stromal and immune cells [105]. In 2003, Hendrix et al. suggested that aggressive cancer cells could transdifferentiate to endothelial cells [106]. Several following reports showed that CSCs from solid cancers can generate endothelial cells that can form functional vessels in tumor tissues. This ability of CSCs was observed in glioblastoma and colorectal CSCs [107,108,109,110]. The hemogenic endothelium produces hematopoietic stem cells not only in the early stages of development but also in the late stage and in the adult, [97] while CSCs have the ability to produce endothelial cells. Thus, theoretically, hematopoietic stem cells could be derived from solid cancer stem cells through hemogenic endothelium in the TME, especially given the strong association between hematopoiesis and angiogenesis. However, no evidence exists for this direct association even that many new pieces of evidence support the vital relationship between hematopoietic progenitors and vascular endothelium [103,104]. Although the ability of CSCs to produce hematopoietic cells has been documented in many studies, little is known about the mechanisms that regulate this event.

In our laboratory, we used the cancer stem cell model that was established from iPSCs to investigate the ability of CSCs to produce hematopoietic cells [111]. Before the study, CSCs were generated by culturing iPSCs in the presence of CM from the BT549 breast cancer cell line cells. iPSCs were converted into CSCs that were tumorigenic and expressed both stemness and CSC markers [29]. We reported that CSCs could produce hematopoietic cells positive for hematopoietic lineage markers and hematopoietic stem cell markers CD34, c.Kit, and Runx1, suggesting that CSCs can be differentiated into hematopoietic progenitors or stem cells. After one month of the injection of these cells into mouse tail veins, cells originating from the injected cells were detected in the circulation blood. Injected cells were also detected in the bone marrow, which suggested that injected cells have hematopoietic stem cell characteristics such as the ability to home into the bone marrow and produce blood cells in the circulation. CSCs were differentiated into macrophage-like cells in the presence of interleukin 3 and stem cell factor. Despite much evidence for hematopoiesis from cancer cells, the direct evidence for the existence of hematopoietic cells from different origins in the TME has not been reported yet to the best of our knowledge [82,83,84,85,86,87,88,89]. Increasing amounts of data support the new hypothesis of the ability of CSCs to differentiate in the TME into different types of hematopoietic cells or progenitors with a homing potential to the bone marrow [82,83,84,85,86,111]. The differentiation mechanisms of CSCs into hematopoietic cells, cytokines, and factors that stimulate differentiation and types of hematopoietic cells that arise from solid CSCs in the TME have not yet been studied. Finally, the question as to why and how is it happening opens a new area of research in the hematopoiesis field.

Further research could elucidate CSCs’ abilities to construct their microenvironments and thereby advance the research focusing on immunotherapy approaches. Identifying the origins of hematopoietic cells will assist in recognizing the cellular and molecular mechanisms of suppressing or activating the immune response against tumors. This could provide new targets for cancer immunotherapy.

Evidence supporting the idea of hematopoiesis from CSCs remains poor and provokes questions about the feasibility of immune cells arising from CSCs. Since many types of immune cells produce tumor-suppressive effects, further questions arise about the possibility of and reasons for CSCs generating immune cells that negatively impact tumor growth. Further doubt exists about the ability of CSCs to produce only immune cells with tumor-suppressive effects. Another key question surrounds CSC models from iPSCs and their relevance to other models’ data. Although several studies have demonstrated the ability of cancer cells with stemness characteristics to provide some hematopoietic cells, as mentioned above, many questions remain to be answered. As such, summarizing efforts that tried to answer parts of these questions is important. These efforts could provide new insights into the origin of hematopoietic cells in the TME according to the plasticity of CSCs, inspiring more research in this field.

## 5. Targeting Hematopoietic Cells in the TME as Cancer Therapies

Cancer therapy outcomes can be improved by enhancing immune system functions using different methods. The effect of cancer drugs and treatments can be boosted by eliminating immune suppressor cells and supporting other cell types that have positive effects on the immune system which could also improve patient survival rates [112]. Targeting molecules that have negative regulation effects on T-cells or natural killer (NK) cells, such as programmed cell death protein (PD-1) and cytotoxic T-lymphocyte antigen-4 (CTLA-4), have shown success in eradicating a wide range of cancers [113]. Other immunotherapy strategies have been proposed to support different types of cancer chemotherapy especially for resistance and metastatic cancer types. These strategies include the activation of NK cells or T-cells [114,115]. Antibodies against PD-1 and LAG-3, a co-inhibitory receptor expressed on T-cells and NK cells, were proposed to restore NK and T-cell functions in cancer patients [116,117]. Targeting both TAMs and MDSCs was found to produce positive effects on survival rates, reducing metastasis and the invasion ability of cancer cells [68]. Antibodies against C−C chemokine ligand 2 (ccl20) and C−C chemokine receptor 2 (ccr2) were found to inhibit the infiltration of TAMs and reduce the tumorigenicity of cancer cells [118]. Sahraei et al. reported that targeting miR-21 in TAMs promotes proinflammatory functions and decreases tumor growth [119]. Another TAM targeting strategy is the inhibition of colony-stimulating factor 1 receptor (CSF1R), which plays crucial roles in TAM survival and recruitment. Antibodies against CSF1R were reported to reduce the number of TAMs in the TME and therefore decrease their tumor-promoting functions [120]. Therapies targeting TAMs could also enhance activation and infiltration of T-cells, which lead to a reduction in tumor growth and metastasis. Recently, many clinical trials have focused on targeting and eliminating MDSCs to enhance the immune responses toward cancers. Inhibition of MDSCs recruitment and activation, or modelling their metabolic pathways are some proposed methods of targeting MDSCs as cancer immunotherapy treatments [70]. All these studies confirmed the importance of hematopoietic cells and their cross-talking and interactions as TME components in cancer treatments.

Although these cells could be promising therapeutic targets, the existence of different subtypes and different activation statuses has created challenges with revealing the exact cell types and mechanisms that should be targeted. In this context, more must be learned about hematopoietic cells in the TME regarding their origins, interactions, mechanisms of proliferation, and activation. Thus, the notion of different origins of hematopoietic cells could contribute to furthering our understanding about the heterogeneous subtypes of hematopoietic cells in the TME.

## 6. Future Perspectives

The relationship between hematopoiesis and solid cancer stem cells is attracting attention, and we now better understand the roles of different types of hematopoietic cells in the TME. However, information is still missing, such as the mechanism and signal pathways for differentiation. Many drugs targeting hematopoietic cells or enhancing the immune responses are currently used in cancer treatment. New research questions are now appearing regarding the effect of these treatments on solid cancer stem cells and their ability to produce hematopoietic cells. The existence of different phenotypes and subtypes of hematopoietic cells in the TME creates complex interactions between them, whereas conditions controlling the balance and dominance of specific subtypes are still unknown despite their pivotal roles in cancer progression. The identification of different subtypes and the interaction between different hematopoietic cells in the TME could provide more insight toward developing more effective cancer treatments. More studies are also needed to explore differences between heterogeneous hematopoietic cell populations in the TME, potentially from different origins, and to assess their relevance to the treatment response.

Recent data also suggest that hematopoietic cells derived from CSCs may pertain to different organs, such as the bone marrow, far from solid cancer primary sites. These cells may also provide peripheral blood with new cells and may shed light on the new era of the plasticity of CSCs and their derived cells as hematopoietic progenitors or stem cells. The concept of normal hematopoietic stem cells driven from solid cancer stem cells is a novel, unfamiliar, and exciting field for future research. Hemoglobin in the tumor should also be examined in the future. Further investigations could provide valuable data for the involvement of these hematopoietic stem cells derived from solid CSCs in the generation of blood and immune cells in response to changes in the TME.
